# 

**Published:** 1895-12-28

**Authors:** 


**Notices of Births, Deaths, and Marriages** are inserted in this column at a charge of 2s. 6d. for an announcement not exceeding four lines.

**Notice to Advertisers and Subscribers.**

All Advertisements, Orders for Copies of The Hospital and Business Communications should be addressed to **THE MANAGER, (Not to The Editor)**, The Hospital, 428, Strand, London, W.C.

The Cost of Subscription, post free, is as follows:—Per week, 2½d.; per quarter, 2s. 8d.; per half-year, 5s. 3d.; per annum, 10s. 6d. Foreign: Per Annum, 15s. 2d.; payable, in all cases, in advance.

### NOTICE TO CORRESPONDENTS.

All MS., Letters, Books for Review, and other matters intended for the Editor should be addressed The Editor, The Lodge, Porchester Square, London, W.

The Editor cannot undertake to return rejected MS., even when accompanied by stamped directed envelope.

**"Burdett's Hospital Annual," 1895,**

Sixth year of publication. 950 pp. Scarlet cloth, gilt lettered. Price 5s. A most complete guide to British, Colonial, Indian, and American Hospitals, Dispensaries, Nursing and Convalescent Institutions, and Asylums. A few complete sets of the preceding five issues of the "Annual" may still be obtained on application to the Publishers, The Scientific Press (Limited), 428, Strand, London, W.C.

**NOTICE.—Vol. XVIII. of "The Hospital" (April to September, 1895), in handsome cloth binding, is now on sale at the Office of this Journal. Price 7s. 6d. Cases for binding the Numbers contained in this Volume, 1s. 6d. Index to Vol. XVIII., 2½d. post free.**

**The Monthly Part for January, 1896, will be on sale January 1, 1896, price 6d., by post, 8d.**

### SCRAPS AND GLEANINGS.

"The Helping Hand" is the name of a new hospital at Peekskill, in America.

Over 1,900 patients have been treated at the Free Eye and Ear Hospital, Southampton, during the past twelve months.

Lord Overton has consented to give a donation of £3,500 to erect and equip an operation-room for the Glasgow Western Infirmary.

The Fishmongers' Company have sent a donation of twenty-five guineas to Queen Charlotte's Lying-in Hospital, Marylebone.

Donations and legacies amounting to £1,416 have been received by the committee of the North Staffordshire Infirmary during the year.

Three well-known French noblemen, the Count de Goyan, the Duke de Rivoli, and the Count de Sinety, have taken the degree of Doctor of Medicine in France.

An entertainment embracing a grand concert and tableaux vivants was held at the Protestant Hall Ballymena, last week, in favour of the funds of the local cottage hospital.

The Royal Holborn Theatre of Varieties was placed at the disposal of the Western Ophthalmic Hospital Committee for a benefit in aid of that institution on the 13th inst.

The performance of the "Overland Route" by the Romany Amateur Dramatic Club in St. George's Hall in aid of the University College Hospital has produced £80 for the funds of the institution.

The total number of cases treated at the Ballymena Cottage Hospital (Belfast) during the year has been 108 intern and 29 extern. A small balance is left on the balance-sheet to begin the New Year with.

A conference of representatives of the various local authorities interested in the provision of an isolation hospital at Droitwich has just been held, to consider particularly the basis of contribution from the various authorities.

Crime, to judge by recent statistics, appears to be on the increase in France. In 1860 the number of minors brought up for trial was 5,400, whereas, in 1891, their number exceeded 7,000, whilst criminals, known to the police, in 1892, exceeded 100,000.

The committee of the Children's Cottage Hospital, Coldash, report well on the working of the institution this year. The entire debt on the building fund has been cleared off, and a steady increase in the number of annual subscriptions is a cause for congratulation.

The Hospital Committee of the Leeds Corporation, at their recent meeting, gave instructions to take immediate steps for the provision of new fever hospitals for the town, the accommodation for infectious diseases being entirely inadequate as at present existing.

During the year the ordinary expenditure of the Aberdeen Hospital for Incurables amounted to £1,666, while the income was only £956; while thus a heavy deficiency is left on the balance. Fortunately, however, some valuable legacies have been received, which have reduced the debt to £428.

A very handsome new staircase for the Dundee Royal Infirmary has been presented by Mr. W. O. Dalgleish, the president. This will be placed near the west entrance of the infirmary, and will replace a staircase which was too narrow and steep for the purpose for which it has been used.

The twenty-fourth annual meeting of the governors and friends of the Northern Counties Hospital for Incurables, Mauldeth, has now been held. The board of management, in their annual report, stated that the institution had just closed another year of usefulness and prosperity.

The managing committee of the Royal Albert Hospital at Devonport, at their annual meeting, recorded an amount of work that had never been equalled in any one year during the existence of the hospital. In response to the special appeal made after the last annual meeting, an appreciable increase of subscriptions and donations had ensued.

The committee of the Clent Convalescent Home have been bestirring themselves to wipe off the liabilities at present existing on the building fund. Accommodation is provided in the home for 30 patients, but, owing to their limited financial resources, the committee have been unable to allow more than 20 patients to be accommodated at one time.

The Cheyne Hospital at Chelsea for Sick and Incurable Children is making a special appeal for further support to enable it to carry on its work. The receipts for the past year have fallen short of the expenditure by a considerable sum, fresh contributions are, therefore, much needed. H.R.H. the Princess of Wales is the President of this charity.

In compliance with the wish of Mr. Henry Gourlay, the directors of the Dundee Infirmary have accepted the proposal to raise the Agnes Gourlay Fund to £600, on the understanding that the income should be applied to the extension of existing objects, and to the setting aside of gifts in money to patients leaving the infirmary and convalescent home.

The need for the additional accommodation provided at the Victoria Infirmary, Glasgow, during the past year has been fully shown, the whole of the beds having been constantly occupied. The handsome gift of £1,050 provided by Mr. Cameron Corbett, M.P., his wife, and sister, for the special purpose of carrying on the dispensary is now practically exhausted.

An appeal is now being issued by the promoters of the 1st Cadet Battalion King's Royal Rifles. This movement is an outcome of the immense effort to keep young lads out of mischief, and to give them habits of discipline and good conduct. Every summer a camp is held by the seaside. Besides this, institutes have been opened in London, which offer many advantages to destitute youths.

A marked feature of the annual report of the Coventry and Warwickshire Hospital is the very considerable increase which has taken place in the number of both in and out patients. The very restricted accommodation for out-patients at present available and the urgent representations of the medical staff have compelled the committee to adopt some course which will have the effect of curtailing the number of such patients.

The annual meeting of subscribers of the Cancer Pavilion and Home at Manchester has just taken place. The committee expressed their gratification at the generous measure of support accorded to them during the past year. The hospital finances show encouraging signs of improvement. A donation of £1,000 from Sir William Agnew and a further donation of £500 from Miss Hannah Buckley have both been added to the endowment fund and invested.

### THE STUDENT OF MEDICINE.

### APPOINTMENTS.

H. H. Back, M.B.Lond., M.R.C.S., appointed Medical Officer of Health to the Aylsham District Council. J. B. Bate, L.S.A., appointed Surgeon in the Royal Mail Steamship Company. R. St. G. S. Bond, M.B., C.M.Edin., appointed Second Assistant Medical Officer to the Metropolitan Asylum, Caterham, Surrey. W. Bowes, M.R.C.S.Eng., appointed Medical Officer for the Sixth District of the East Ashford Union. E. P. Dickin, M.B., C.M.Edin., appointed House Surgeon to the Northampton Infirmary. A. Eddowes, M.D.Edin., appointed Physician on the staff of the St. George's and St. James Dispensary, King Street, Regent Street, W. A. G. Foljambe Foster, M.B., C.M.Edin., appointed Assistant House Surgeon to the Northampton Infirmary. F. Harvey, M.R.C.S.Eng., L.S.A., appointed Medical Officer of Health to the Padstow Urban Council. J. A. Hogg, M.R.C.S.Eng., L.R.C.P.Lond., appointed Medical Officer for the Shardlow District of the Shardlow Union. W. M. Jennings, M.B., appointed Medical Officer for the Kentchurch District and the Workhouse of Abbeydore Union. G. R. Leighton, M.B., C.M., L.R.C.P. and S.Eng., L.F.P.S.G., appointed Medical Officer to the Skenfrith District of the Monmouth Union. F. W. Lewis, M.R.C.S., L.R.C.P.Lond., appointed Senior House Surgeon to the General Infirmary at Gloucester and the Gloucestershire Eye Institution. T. D. Lister, M.D., B.S.Lond., F.R.C.S.Eng., appointed Honorary Surgeon to the Westminster General Dispensary. E. P. Manby, M.B., M.R.C.S.Eng., appointed Assistant Medical Officer of Health for Liverpool. T. Moore, M.R.C.S Eng., L.M., appointed Medical Officer of Health for the Stockport Rural District. J. O'Connell, L.R.C.P., L.R.C.S., appointed Senior Assistant Medical Officer at the Toxteth Park Workhouse. J. Patrick, M.B., appointed Assistant Medical Officer to the Belfast Lunatic Asylum. C. J. Perrott, L.R.C.P., L.R.C.S.I., appointed Medical Officer of Health to the Kingswood Urban Council. T. W. Richardson, M.R.C.S.Eng., appointed District Medical Officer of the Beckham Union. H. Stanley, M.B., appointed Medical Officer for the Fifth District of the East Ashford Union. S. Stephenson, M.B., F.R.C.S.E., appointed Ophthalmic Surgeon to the North-Eastern Hospital for Children, Hackney Road, Shoreditch. H. E. Thompson, M.R.C.S., L.R.C.P.Lond., appointed Assistant House Surgeon to the General Infirmary at Gloucester and the Gloucestershire Eye Infirmary. T. M. Tibbetts, M.B.Lond., M.R.C.S.Eng., appointed Medical Officer of Health for the Quarry Bank Urban District. J. P. Wagstaff, L.R.C.P.Lond., M.R.C.S., appointed Medical Officer for the Pelham District of the Bishop Stortford Union. Dr. E. W. Walker, appointed Medical Officer of Health for the Shoeburyness Urban District. L. H. Walsh, L.R.C.P.Lond., M.R.C.S., appointed Resident Medical Officer to the Royal Mineral Water Hospital, Bath.

### VACANCIES.

The following vacancies are announced this week, the amount of salary in each case being given after the name of the institution:—

### Resident Medical Officers.

Bridgnorth and South Shropshire Infirmary.—House Surgeon; doubly qualified. Salary £80 per annum, with board and lodging in the infirmary. Appointment for one year, but eligible for re-election. Applications to the Honorary Secretary, Oldbury Rectory, Bridgnorth, by December 31st.

Brighton and Hove Lying-in Institution and Hospital for Women, 76, West Street, Brighton.—House Surgeon; unmarried, and under 30 years of age. Salary £80 per annum, with furnished quarters and board, gas, coals, and attendance. Applications to the Honorary Secretary before December 31st.

Bristol Incorporation.—Medical Officer for the Workhouse at Stapleton; doubly qualified. Salary £250 per annum, with residence and rates and taxes free, together with vaccination fees. Applications to J. J. Simpson, Clerk to the Guardians, St. Peter's Hospital, Bristol, by December 31st.

Chorlton upon-Medlock Dispensary, Manchester.—Resident House-Surgeon; doubly qualified; unmarried. Salary £100 per annum, with furnished rooms and attendance. Applications to the Secretary before December 28th.

County Borough of Cardiff.—Resident Medical Officer of the Brompton Hospital for Infectious Diseases; unmarried. Appointment for one year. Salary £50 per annum, with board (without stimulants) and residence in the hospital. Applications to Dr. Walford, Medical Officer of Health, Town Hall, Cardiff, by January 1st, 1896.

Devon County Asylum.—Assistant Medical Officer; single. Salary £120 per annum, with board, lodging, and washing. Applications to Arthur E. Ward, Clerk to the Visitors, 9, Bedford Circus, Exeter, by December 31st.

Taunton and Somerset Hospital.—Assistant House Surgeon. Appointment for six months, without salary, but board, washing, and lodging in the institution provided. Applications, endorsed "Assistant House Surgeon," to J. H. Biddulph Pinchard, Secretary, 13, Hammet Street, Taunton, by December 31st.

Westminster General Dispensary, Gerrard Street, Soho, W.—Resident Medical Officer. Applications to the Secretary by December 30th.

Wolverhampton and Staffordshire General Hospital, Wolverhampton.—Resident Assistant. Appointment for six months. Board, lodging, and washing provided. Applications, inscribed "Application for Resident Assistant," to the Chairman of the Medical Committee by December 30th.

York County Hospital.—Assistant House Surgeon; doubly qualified. Salary £60 per annum, with board, rooms, washing, &c. Applications to Fred. W. Howell, Secretary and Manager, by January 1st, 1896.

### Non-Resident Medical Officers.

Central London Throat, Nose, and Ear Hospital, Gray's Inn Road, W.C.—Assistant Registrars. Applications to Richard Kershaw, Secretary, by January 11th, 1896.

Dental Hospital for London, Leicester Square, W.C.—Assistant Dental Surgeon; must be L.D.S. Applications to J. Francis Pink, Secretary, by January 6th.

Dental Hospital for London and London School of Dental Surgery, Leicester Square, W.C. — Demonstrator. Honorarium £50 per annum. Applications to J. Francis Pink, Secretary, by January 6th.

Hospital for Consumption and Diseases of the Chest, Brompton.—Four Clinical Assistants in the Out-Patient Department, and Clinical Clerks to the In-Patient Physicians. Applications to the Secretary by December 31st.

Owens College, Manchester.—Junior Demonstratorship in Physiology and Histology. Salary £100 per annum. Applications to the Registrar by January 4th, 1896.

Poplar and Stepney Sick Asylum District.—Second Assistant Medical Officer for the Asylum at Bromley, Middlesex. Salary £80 per annum, increasing £10 yearly to £100. Applications, on forms provided, to be sent to Robert Foskett, Clerk to the Managers, Bromley, Middlesex, E., by January 2nd.

Samaritan Free Hospital for Women and Children, Marylebone Road, N.W.—Surgeon to the Out-patient Department. Applications to the Secretary, George Scudamore, by January 7th, 1896.

### "THE HOSPITAL"

AN INDEPENDENT JOURNAL OF

**The Medical Sciences**

AND

**Hospital Administration,**

WITH WHICH IS ISSUED WEEKLY

**A SPECIAL**

### SUPPLEMENT FOR NURSES.

PUBLISHED EVERY SATURDAY.

PRICE TWOPENCE.

Matrons, Sisters, or Nurses will find in The Hospital a chronicle of the latest Nursing News, Practical Articles of the highest value to those who wish to keep themselves abreast with the times, and brightly written articles from Nurses all over the world.

**SUBSCRIPTIONS CAN COMMENCE AT ANY TIME.**

### TERMS OF SUBSCRIPTION.

### WEEKLY ISSUE.

| | Great Britain. | | Foreign and Colonial. |
|---|---|---|---|
| For One Year ... | 10s. 6d. | ... | 15s. 2d. |
| For Six Months | 5s. 3d. | ... | 7s. 7d. |
| For Three Months... | 2s. 8d. | ... | 3s. 10d. |

### MONTHLY PARTS.

| | | Post Free to any Part of the World. |
|---|---|---|
| For One Year | ... ... | 8s. 6d. |
| For Six Months | ... ... | 4s. 3d. |
| For Three Months ... | ... | 2s. 2d. |

Postal Orders and Cheques should be made payable to "The Scientific Press, Limited."

N.B.—In order to prevent delay, all business communications should be addressed to "The Manager" only, and not to any person individually.

### CHANGE OF ADDRESS.

In event of change of address, notice should be forwarded to the Publishers by the Saturday previous to date of publication, and the old address should also be given.

"The Hospital" can be obtained of all Booksellers and Newsagents in the United Kingdom, and at all the Railway Stalls, of Messrs. W. H. Smith and Son; and also from the following Agents in the Colonies and Abroad:—

Paris: Librairie Galignani, 224, Rue de Rivoli.
Berlin: Messrs. A. Asher and Co.
Leipsig: Mr. F. A. Brockhaus.
New York: The International News Co.
Montreal: The Montreal News Co.
Toronto: The Toronto News Co.
Melbourne, Sydney, Adelaide, and New Zealand: Messrs. Gordon and Gotch.
India: At all the Railway Bookstalls and Agencies of Messrs. A. H. Wheeler and Co.
South Africa: Messrs. Juta, Cape Town.

Publishing Offices: 428, Strand, London, W.C.

London: THE SCIENTIFIC PRESS (Limited), 428, Strand, W.C.

*In the Press. Price 5s.*

### BURDETT'S OFFICIAL NURSING DIRECTORY

A DIRECTORY, NOT A REGISTER.

THE HOSPITAL states: "It is well to bear in mind the difference and distinction which exists between a register and a directory. A register implies rights, and suggests that those who are not upon it are in some way or another inferior beings. But a directory is another matter. A directory gives as rights; insertion in it implies no adherence to any particular party or any particular opinion, and in no way interferes with entry on the register of any association, or the list of any institute, to which the individual may belong. All that a directory professes to do is to give information. The 'Medical Register' is not a mere list of qualified medical men; it is admission to the Register, which itself makes him qualified. For years, perhaps even now, 'Churchill's Medical Directory' contained the names of medical men who, so far as public appointments were concerned, were unqualified; it also contains the names of homœopathists, whom four out of every five medical men will not meet; and it certainly contains the names of many for whose moral character we should not like to vouch. But it gives full and accurate information about them all, and for the sake of that information it is constantly consulted both by the public and the doctors."

WHAT NURSES NEED.

"At present there is no Register for the nursing profession giving any legal status. Such a thing may come or it may not; but in either case there is no reason why there should not be a Directory, giving such information about the career of each nurse as both the public and the profession want; information, by the by, which no legal Register would be likely to insert. The 'Medical Register' is a bald and meagre thing compared with the 'Medical Directory.' What Burdett's 'Official Nursing Directory' proposes to do is to supply the want of a directory giving information. It will give no rights, it will imply no partisanship, it will give no guarantee except that what it prints is true, and is derived from official sources."

ACCEPTABLE TO ALL NURSES.

"This ought to be enough to make it acceptable to the public, and every nurse ought to make sure that, whatever list or register she may be on, her name shall also appear on Burdett's 'Official Directory.'" Applications for forms should be made at once to the Editor, "Burdett's Official Nursing Directory," 428, Strand, W.C.

**The above work will be ready early in the coming year, and those Nurses who have not already sent in their names should at once apply for a form, which can be obtained upon application to the Publishers,**

THE SCIENTIFIC PRESS (Lim.), 428, Strand, London, W.C.

### ROYAL NATIONAL PENSION FUND FOR NURSES.

*President*—**H.R.H. THE PRINCESS OF WALES.**

*Chairman*—WALTER H. BURNS, Esq. *Deputy-Chairman*—HENRY C. BURDETT, Esq.

**PENSIONS. SICKNESS. ACCIDENT.**

### Total Invested Funds, £250,000.

Nurses are invited to join the Fund on account of the substantial and exceptional advantages which offers them and which *they cannot obtain elsewhere*. The following are the chief points:—

1. **The Fund is Mutual and essentially Co-operative.**
2. **Easy Payment of Premiums.** Nurses can pay their premiums monthly or otherwise as best suits their convenience.
3. **The Fund is open to every Nurse.** Nurses can assure for Pensions of any amount commencing at any age.
4. **An Investment and Savings Bank.** Those entering under the returnable scale can have their premiums returned to them with compound interest, less a small deduction for working expenses. Interest allowed on deposits.
5. **Additions to Pensions.** Besides the Pension entered for, substantial additions thereto may be anticipated in the shape of bonuses.
6. **Sickness and Accident Assurance.** Policies are issued in connection with Pension policies assuring 10s., 15s., or 20s. a week in cases of incapacity from work through sickness or accident.

Full particulars to be obtained from **THE SECRETARY,**

ROYAL NATIONAL PENSION FUND FOR NURSES,

28, FINSBURY PAVEMENT, LONDON, E.C.

### STANDARD TEXT-BOOKS ON NURSING, &c.

*Just Published, 8th Edition. Greatly enlarged and thoroughly revised. Crown 8vo., cloth gilt, 3s. 6d.*

### NURSING: ITS THEORY AND PRACTICE,

BEING A

### COMPLETE TEXT-BOOK OF MEDICAL, SURGICAL, AND MONTHLY NURSING.

**By PERCY G. LEWIS, M.D., M.R.C.S., L.S.A., &c., &c.**

To this edition have been added three chapters on Monthly Nursing, together with additions to the chapters on Blood Diseases, on Antiseptics, on the Nursing of Children, and on Private Nursing. Under these headings will be found a short account of the new Aseptic Treatment, the Antitoxin and Serum Treatments, and the Treatment by Thyroid Extracts.

These additions, combined with the revision throughout of the text, render this work the best and most complete Text-book on Nursing in the market, and strengthen its position as a Classic in the Training Schools of the World.

Just Published. With numerous Illustrations, demy 8vo., blue buckram, gilt, 3s. 6d.

### DIET IN SICKNESS AND IN HEALTH.

**By Mrs. ERNEST HART,**

*Formerly Student of the Faculty of Medicine of Paris, and of the London School of Medicine for Women.*

**With an Introduction by Sir HENRY THOMPSON, F.R.C.S., M.B. Lond.**

Sir Henry Thompson, in his Introduction, says: "I do not hesitate to express my opinion that the present volume forms a handbook to the subject, which will not only interest the dietetic student, but offer him, within its modest compass, a more complete epitome thereof than any work which has yet come under my notice."

"The importance of the subject to which Mrs. Ernest Hart has devoted this volume will not be disputed by any practical medical man. As to how Mrs. Ernest Hart has discharged her self-imposed task we prefer to quote from the introduction the words of Sir Henry Thompson, than whom there could be no more competent critic of a subject which he has himself so much illuminated by his admirable essays."—"British Medical Journal."

"Medical men will find much of the information given very useful in daily practice. The chapters dealing with the principles which underlie scientific dietetics are characterised by common sense, and are replete with practical suggestions and directions. The chapter on the alimentation of diabetics is of special excellence. Not only are the general rules of diet in this morbid condition carefully discussed and explained, but we have a complete scheme of diet with *menus* for a whole week, showing what a vast amount can be done to palliate the irksomeness of the restrictions by a little ingenuity, forethought, and resource. On the whole we endorse Sir Henry Thompson's recommendation of the book as 'supplying an important want in our educational literature.'"—"The Medical Press."

Second and Revised Edition. Demy 16mo., suitable for the apron pocket, handsomely bound in cloth, 140 pp. Price 2s.; in handsome leather, gilt, price 2s. 6d. net.

**THE NURSES' DICTIONARY OF MEDICAL TERMS AND NURSING TREATMENT.**—Compiled for the use of Nurses by HONNOR MORTEN. Containing descriptions of the principal Medical and Nursing Terms and Abbreviations, Instruments, Drugs, Diseases, Accidents, Treatments, Physiological Names, Operations, Foods, Appliances, &c., &c., encountered in the Ward or Sick-room.

200 pp., Crown 8vo., cloth, 3s. 6d. Profusely Illustrated with Original Drawings, with list of Instruments required, and a Glossary of Terms.

**OPHTHALMIC NURSING.** By SYDNEY STEPHENSON, M.B., F.R.C.S.E., Surgeon to the Ophthalmic School, Hanwell, W., &c., &c. This is a valuable contribution to Nursing literature on a subject hitherto never handled with special reference to Nursing. On account of its compact and clear arrangement, and its numerous illustrations, it is specially recommended to the use of the Nursing Profession.

Second Edition, with a New Chapter and several Coloured Plates Illustrative of Feeding Bottles used in ancient times, &c. Crown 8vo., imitation cloth, 5s. With 10 plates and many Illustrations in the Text, and Fac-simile Autograph Letters from the late Sir Andrew Clark, M. Pasteur, &c.

**INFANT FEEDING BY ARTIFICIAL MEANS**: A Scientific and Practical Treatise on the Dietetics of Infancy. By S. H. SADLER, Author of "Suggestions to Mothers," "Management of Children," "Education."

Crown 8vo., cloth gilt, Illustrated. price 5s.

**HELPS IN SICKNESS AND TO HEALTH**: Where to Go and What to Do. Being a Guide to Home Nursing and a Handbook to Health in the Habitation, the Nursery, the School-room, and the Person, with a chapter on Pleasure and Health Resorts. By HENRY C. BURDETT, Author of "Hospitals and Asylums of the World," "Pay Hospitals of the World," "Cottage Hospitals, General, Fever, and Convalescent," &c., &c.

"It would be difficult to find one which should be more welcome in a household than this unpretending but most useful book."—*The Times.*

Important New Handbook of Midwifery. Handsomely bound in leather, 260 pp., profusely illustrated, price 6s.

**A PRACTICAL HAND-BOOK OF MIDWIFERY.** By FRANCIS W. N. HAULTAIN, M.D. A Practical Manual produced in a portable and convenient form for reference.

Third Edition, enlarged and partly re-written, crown 8vo., cloth gilt, about 200 pp., price 2s. 6d.

**HOSPITAL SISTERS AND THEIR DUTIES.** By EVA C. E. LUCKES, Matron to the London Hospital. The favourable reception accorded to the first two editions of this useful and interesting work, and the regular demand for copies of it, encourage the publishers to place this third edition, greatly improved and enlarged, before the Institutions. The experience and position of its author entitle it to authority, and every Matron, Sister, or Nurse who aspires to become a Sister should read the advice and information contained in its pages.

Crown 8vo., 500 pp., Second Edition, 7 Plates, 18 Illustrations, Charts, &c., price 7s. 6d. net.

**NURSING.** By ISABEL ADAMS HAMPTON.

"It is not too much to say that in no single volume yet published has the subject been so scientifically, carefully, and completely treated as it has been by Miss Hampton. . . . Whether as a teaching manual or as a book of reference, the thoroughly practical instruction which it conveys is sure to obtain a wide circulation."—*The Hospital.*

Crown 8vo., cloth gilt, containing many Plates and Illustrations, price 2s. 6d.

**SPINAL CURVATURE AND AWKWARD DEPORTMENT**: Their Causes and Prevention in Children. Dr. GEORGE MULLER, Professor of Medicine and Orthopædics, Berlin. English Edition, edited and adapted by RICHARD GREENE, F.R.C.P.

"An able essay upon the effect produced by exercise, attitude, and movement upon the growth and development of the body. He further gives directions which any intelligent parent or teacher could carry out with the help of very simple apparatus, showing how to avoid or, in early cases, to cure certain malformations which, in the majority of cases, arise either from the neglect of very simple hygienic rules or from carelessness."—*Daily Chronicle.*

LONDON: THE SCIENTIFIC PRESS (LIMITED), 428, STRAND, W.C.

### PAMPHLETS ON NURSING.

No. 1, price 1½d. per copy, or 1s. per dozen.

**THE ADVANTAGES AND PRIVILEGES OF A TRAINED DISTRICT NURSE, AND THE DUTIES OF THE DISTRICT TOWARDS HER.**

By HENRY C. BURDETT.

No. 2, price 1½d. per copy, or 1s. per dozen.

**A GREAT MOVEMENT—THE NURSES' CO-OPERATION.**

By Mrs. FURLEY SMITH.

London: THE SCIENTIFIC PRESS (LIMITED), 428, STRAND, W.C.

